# Follow-up of HTLV-1 positive individuals in the GIPH cohort (1997-2013): Proviral load was not a prognostic marker for HAM/TSP

**DOI:** 10.1186/1742-4690-11-S1-P23

**Published:** 2014-01-07

**Authors:** Poliane C Gonçalves, Gabriela S Freitas, Luiz CF Romanelli, Fernando A Proietti, Anna B Carneiro-Proietti, Marina L Martins

**Affiliations:** 1 GIPH (Interdisciplinary HTLV Research Group); Hemominas, Belo Horizonte, Minas Gerais, Brazil; 2 Faculdade da Saúde e Ecologia Humana (FASEH), Vespasiano, Minas Gerais, Brazil

## Background

HTLV-1 proviral load (PVL) is considered a risk marker for diseases.

## Methods

Quantification of HTLV-1 PVL was performed in 151 samples of 38 asymptomatic carriers (AC) collected at different times during follow-up (6.1 to 14.8 years, mean 10) and in samples of five individuals who developed HAM/TSP during follow-up (2.6 to 11.3 years, mean 7.2). We used SYBR Green and number of proviral copies/10,000 cells. Fluctuation of proviral load level was defined at 0.5 log or more.

## Results

PVL was stable in 52.6% (20/38) and floated in 47.4% (18/38) subjects. In AC, the median of PVL in the 1st sample was 85 and in the last 59 (p = 0.59). Among those individuals with low PVL who showed fluctuation, it remained low (£1%) in 77.8%. In 60% with high PVL who showed fluctuation, it remained high during follow-up. 10 patients developed HAM/TSP during the follow-up, and PVL was quantified before and after in 5 cases. Median of PVL in the 1st sample was 445, and in the last sample 98 (p = 0.56). In all cases, PVL was higher in the asymptomatic period, declining after onset of HAM/TSP.

## Conclusions

PVL reaches a plateau, characteristic of each individual; high PVL appears to be followed by decrease and stabilization in lower levels. Although PVL is supposedly a risk marker for HAM/TSP, it had modest prognostic value in our cohort; changes in clinical status and PVL did not coincide, besides occurrence of high stable PVL in AC. Hemominas/FAPEMIG/DECIT/MS.

